# Pilot Study: The Relationship between Foot Posture and Movement Quality in Non-Professional Male Football Players

**DOI:** 10.3390/life13071574

**Published:** 2023-07-17

**Authors:** José Algaba-Del-Castillo, Aurora Castro-Méndez, Ana Juana Pérez-Belloso, José Gabriel Garrido-Barragán, Alberto Aguilar Sánchez, Manuel Coheña-Jiménez

**Affiliations:** 1 Podiatry Department, University of Seville, 41009 Seville, Spain; algaba@us.es (J.A.-D.-C.); aperez30@us.es (A.J.P.-B.); mcohena@us.es (M.C.-J.); 2 Independent Researchers, Avenida de Los Pinos 1, Montequinto, 41089 Seville, Spain; josegabrielgarrido@outlook.es (J.G.G.-B.); badaspodologia@gmail.com (A.A.S.)

**Keywords:** pronation, supination, movement quality, foot

## Abstract

(1) Background: This study focusses on establishing the relationship between quality of movement (based on the functional movement screen, or FMS) and foot posture (based on the foot posture index, or FPI-6). We hypothesised that a poor FMS test score may be derived from the foot position in the space measured by FPI-6. (2) Methods: a quasi-experimental and cross-sectional study was designed to analyse foot posture in 30 healthy football players, using the foot posture index and the functional movement screen. (3) Results: No significant relationships were found between movement quality and foot posture. Poor movement quality is present in more than half of all foot positions, supination, pronation, and neutral. Good quality seems to be more associated with a neutral foot position (23.3%) and supinated (16.6%) than a pronated foot position (6.6%). (4) Conclusions: this study found no relationship between the two tests; therefore, we cannot demonstrate that foot posture is relevant in the quality of the movement of the football players studied.

## 1. Introduction

Musculoskeletal injuries are common occurrences during physical exercise, especially among people with a low level of training [1,2,3,4]. Furthermore, different sports have a specific movement pattern that can estimate the risk of injury for athletes. These injuries can have a significant impact on athletic performance and overall well-being [5]. Recent studies have shed light on the influence of foot morphology on plantar pressure patterns, gait, and running mechanics [6,7,8]. The foot plays a crucial role in supporting the body’s weight, adapting to ground loads, and facilitating various sporting gestures, such as jumping, running, changing direction, lateral displacements, shooting, and landing from jumps, particularly in sports like football [6]. To cope with ground reaction forces, the knee of the kinetic chain, as a joint with limited range of motion, depends on the mechanical behaviour of the hip and ankle to mitigate and distribute forces appropriately [9]. Specifically, it is believed that the type of foot an athlete has can affect plantar loads during sporting activities [7]. Consequently, joint failure can cause injury in athletes, as repetitive tasks performed in various sports can influence the risk factors for injury. Foot posture and its variations have been suggested to influence lower limb function and contribute to the development of injuries [8,10]. For example, a common factor in the development of knee injuries is the presence of a dynamic valgus of the lower extremity, including weak hip musculature and hindfoot eversion in pronated valgus feet [10]. Therefore, movement quality has been shown to be related to morphological parameters, such as body composition or repetition speed in soccer players [11]. The study by Sannicandro et al. showed an association between better foot movement screen scores and higher or lower extremity power among professional soccer players [12].

The foot is a fundamental element in maintaining postural stability, adapting to ground loads, and significantly influencing the sporting gestures used in football: jumping, running, changing direction speed, lateral displacements, shooting, and landing on the jump [6]. Soccer is a sport in which weight bearing is significant. Although there is no consensus in the literature on the influence of foot type and risk of injury, these plantar loads are influenced by the type of foot that athletes have [7]. Some studies have highlighted that the repetitive use of sporty gestures in a poor manner, associated with a foot type that is far from “normal”, is a risk factor for the development of injuries in the athlete [7,8,13]. The development of different skin lesions on the foot or deformities of them can be precursors to muscular injuries [14]. Although it is possible that the dynamic interaction of several factors can lead to a higher or lower likelihood of injury, foot posture is one of the most important, as variations in foot posture are suggested to influence lower limb function [15,16,17].

To assess movement quality, various tools and tests have been developed. The functional movement screen (FMS) is a tool used for the objective detection of body mobility, stability, and movement control in athletes, and evaluates the quality of fundamental movement patterns to identify limitations or asymmetries in individuals [18,19]. A meta-analysis indicates the ICC for intra-rater reliability was 0.81 (95% CI, 0.69–0.92), and for interrater reliability was 0.81 (95% CI, 0.70–0.92) [17]. It has gained popularity among professional and university sports teams, as well as in tactical professions such as the military, firefighting, and law enforcement [20]. FMS makes it possible to assess dynamic neuromuscular stabilisation training versus general physical training. Using the FMS, trainers and coaches can pinpoint areas of weakness or dysfunction in athletes’ movement patterns, helping to tailor training programmes [21].

In addition to the FMS, the foot posture index (FPI-6) has been used to assess foot position [22]. Numerous studies have used this validated tool to establish the relationship between the presence of musculoskeletal disorders and different foot positions [23,24]. The foot is a complex structure that exhibits both rigidity and flexibility. It must provide stability during weight-bearing activities while also adapting to the environment. However, deviations from the optimal foot posture in the three planes of space can lead to various musculoskeletal disorders such as Achilles tendinopathy, plantar fasciitis, or back pain [25].

Both are tests commonly used in scientific research. FMS was developed to assess the fundamental movement patterns of an individual and to be a predictor of injury [26,27]. The use of FMS for the assessment of muscular flexibility provides interesting information in conjunction with complementary tests of active flexibility, helping to predict the risk of injury in athletes [28]. By examining the scores obtained from these evaluations, practitioners can classify athletes. FMS allows for the classification of movement quality as perfect (=21), optimal/good (14–20), and poor (<14); on the other hand, FPI-6 allows to classify the foot position as pronated (+6 to +12), neutral (0 to +5), or supinated (−1 to −12) [29,30,31,32]. However, to date, no study has explored the relationship between the scores obtained from these two tests or established specific risk parameters to use in preventive training programmes.

Therefore, the main objective of this study was to investigate the potential correlation between movement quality, as assessed by the FMS, and foot position, as evaluated by the FPI, among young non-professional football players. It was hypothesised that a low score on the FMS test may indicate an atypical foot position measured by the FPI. By examining this relationship, researchers aim to contribute to the existing body of knowledge on injury prevention and rehabilitation strategies for athletes.

## 2. Materials and Methods

### 2.1. Study Design and Participants

A descriptive cross-sectional study was designed. The study was carried out with non-professional football players from two different football teams from two provinces of Andalusia (Spain). Both Unión Deportiva Pilas (UDP) from Seville and Atlético Onubense (ATCO) from Huelva are teams integrated in the First Andalusian Division of amateur football. The players were measured during the first week of the season, with a training volume of four sessions per week (2.5 h in each session). Data were collected in the Clinical Podiatry Area of the University of Seville. The Clinical Podiatry Area gave permission to carry out the study.

The eligibility criteria were as follows: inclusion criteria were men aged 18–34 years, playing amateur football, and giving their written consent before participating. Exclusion criteria were as follows. Participants were excluded if they had any of the following situations: muscle injuries, participants in the recovery process, having had any musculoskeletal limitation during childhood, a history of lower extremity surgery, playing a sport other than football, goalkeepers, and participants with different foot position values for each foot (the global FPI of both feet is a reference value) [33,34].

### 2.2. Procedures and Assessments

After verbally informing all volunteer participants who met the inclusion criteria for participation in the study, they received an information document about the study and the informed consent form to sign and return. Personal affiliation data, name, date of birth, weight, and height, were collected. All participants were barefoot and evaluated using the Tanita BC 731W (Tanita Corp., Tokyo, Japan). The 36 football players were scheduled to begin health evaluations before the start of the season, with the aim of determining their quality of movement status prior to the competition period. Finally, players who met the eligibility criteria were assessed with both the FMS and the FPI. Both tests were carried out at the same closed site and on the same day for each player. When conducting the study in a closed environment and on the same day, the consistency of conditions was ensured for all participants. This helped minimise any external variability that could affect the results. Additionally, by assessing both the FMS and the FPI, a comprehensive picture of each player’s movement status and foot posture was obtained.

**Figure 1:**
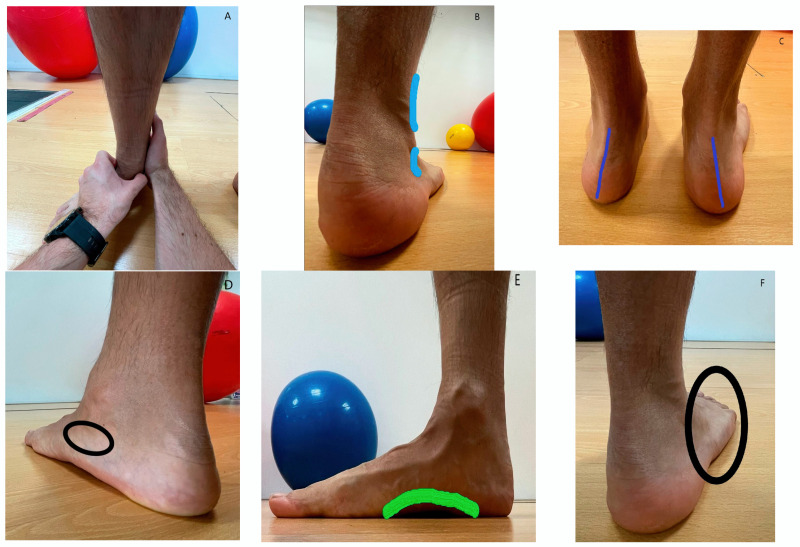
Foot posture index.

First, the participants were scanned and assessed using the foot posture index (FPI-6) for both feet. This foot posture index is a validated measurement method used in numerous studies to quantify foot morphology, which allows differentiation between 3 foot types or foot positions: supinated position, pronated position, or neutral position. It is particularly relevant in the context of football players, as foot posture can influence movement quality and sports performance. By evaluating foot posture, healthcare professionals can identify potential misalignments or issues that could affect the function and performance of the player. For this purpose, it evaluates the participant’s foot with six criteria and gives a score that is progressively added up. The criteria are as follows (Figure 1): palpation of the talar head (A); supralateral and infra-lateral malleolus curvature (B); position of the frontal plane of the calcaneus (C); prominence in the neutral joint of the talar region (D); congruence of the medial longitudinal arch (E); and abduction/adduction of the forefoot over the hindfoot (F). Each criterion is evaluated from 2 to +2, with a range of −12 to +12. The total score can range from the maximum that would indicate a pronated foot position (+6 to +12), to an intermediate score (0 to +5) that corresponds to a neutral foot position, and the lowest possible score, which would be 12 to 1 and would be considered a supinated foot position [35,36,37,38].

**Figure 2:**
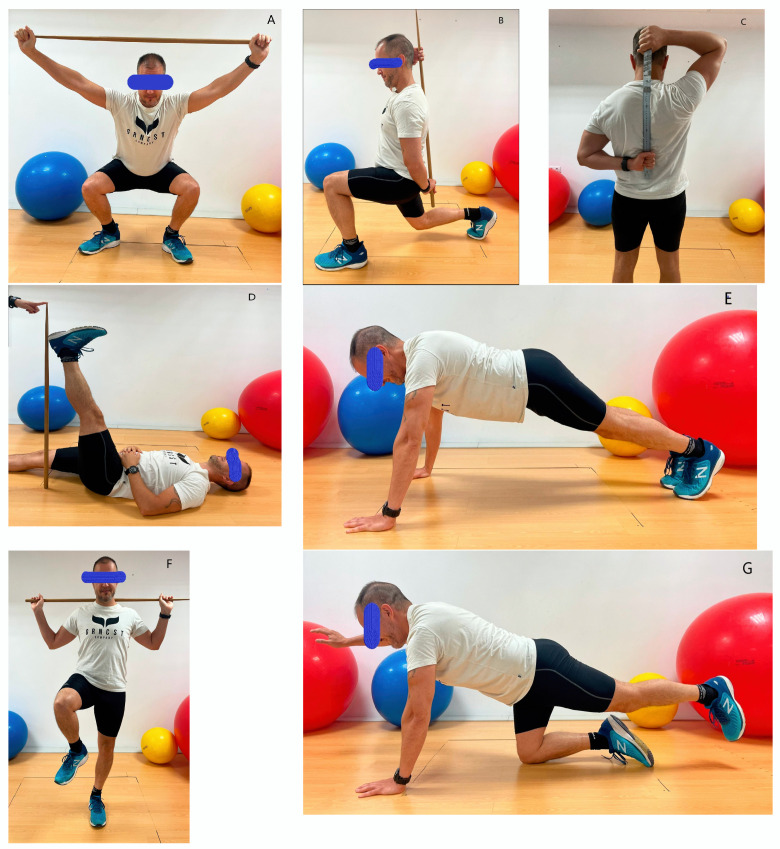
Functional movement screening test. Scoring criteria on a 0–3 rating scale.

The FMS or functional movement screen is a tool used for the objective detection of body mobility, stability, and movement control of football players and evaluates basic movement patterns including seven tests (Figure 2): deep squat (A), inline lunge (B), shoulder mobility assessment (C), active straight-leg raise (D), trunk stability push-up (E), hurdle step (F), rotary stability (G). These tests can be grouped into three subcategories: mobility (A, D, E, G), stability (B, F), and balance (C). This tool has excellent inter- and intra-observer reliability. The intraclass correlation coefficient for intra-observer reliability was 0.81 (95% CI) and 0.81 (95% CI) for inter-observer reliability [39,40].

**Figure 3:**
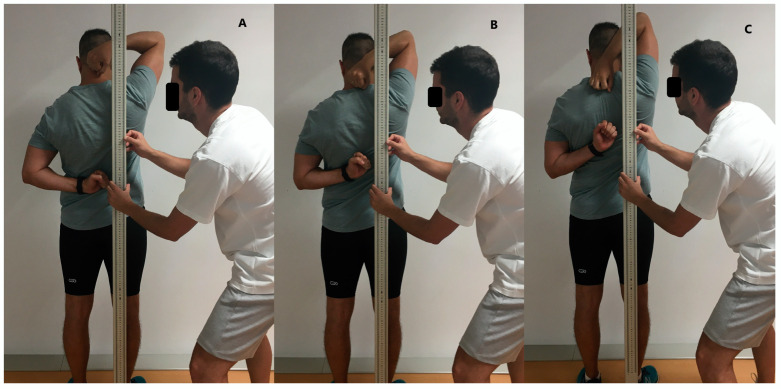
Functional movement screening test. Shoulder mobility assessment: (**A**) rating scale: 1; (**B**) rating scale: 2; (**C**) rating scale: 3.

Each test is evaluated on a scale of 0–3, and each individual performed each task three times, recording the lowest score achieved (Figure 3). In asymmetric tests, both limbs were assessed separately and the right limb was always assessed [41,42].

**Figure 4:**
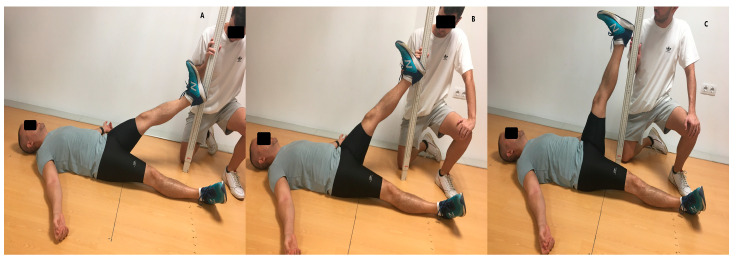
Functional movement screening test. Active straight leg raise: (**A**) rating scale: 1; (**B**) rating scale: 2; (**C**) rating scale: 3.

A score of 3 means that the participant has a correct movement pattern, a score of 2 means that the movement occurs with compensation but not in the correct way, and a score of 1 means that he could not perform the task in a coordinated or correct way, and 0 means that the subject has pain at any time during the movement (Figure 4). The total result of the sum of all points ranges from 0 to 21 points and allows the classification of the movement quality as perfect = 21, optimal 14–20, and poor <14 [43,44].

The collected data were then analysed to explore the potential relationship between FMS and FPI scores. Data were processed in IBM SPSS v.25 for the statistical study. Quantitative variables were expressed as averages and standard deviations, and qualitative (non-numerical) variables were expressed as frequencies and percentages. Cross-tabulations of the FMS and FPI tests were used to differentiate the relationships between variables [45]. Pearson’s chi-square test was used to test for this association. In all hypothesis tests, a significance level of *p* < 0.05 was considered.

### 2.3. Ethical Considerations

This study was approved by the Biomedical Research Ethics Committee (code number: 1374-N-19) of the Junta de Andalucía (Andalusian Regional Government). The ethical standards for human research stipulated in the Declaration of Helsinki (World Medical Association), in the Council of Europe Convention on Human Rights and Biomedicine, in the UNESCO Universal Declaration on the Human Genome and Human Rights, and in similar institutional declarations were observed at all times. This research follows the rules established in CONSORT. The study is authorised by the Clinical Podiatry Unit of the University of Seville. Participation was voluntary. No incentives were offered for participation in the study.

## 3. Results

### 3.1. Sample Characteristics

The basic data for the population studied were as follows: The final sample size was 30 male participants and professional football players. The mean age was 22 ± 3.45 years (range 31–19), the mean body weight was 73.12 ± 7.13 kg (median ± SD), and the mean height was 1.79 ± 0.359 m (median ± SD). The FMS was 14.3 ± 2.35 (median ± SD) (range 10–19), and the mean FPI was 0.90 ± 2.65 for the left foot and 0.96 ± 2.70 for the right foot.

### 3.2. Descriptive and Inferential Data

According to the FPI, half of the participants presented a neutral foot position, followed by supination. The pronated foot position was only present in four participants and none of them had hyperpronation of the foot. Regarding movement quality according to FMS, more than half of the participants had poor movement quality and 14 participants managed to obtain optimal quality scores. None achieved a perfect quality score (Table 1).

The descriptive data cross-checked between the two scores does not establish a strong correlation. The data are presented in Table 2.

After comparing the different positions of the foot and the movements of the participants, the results showed that the different positions of the feet in supination, pronation, and neutral were not statistically significant (X^2^ = 0.024, df = 2, *p* = 0.988). Therefore, a relationship between the variables was not established.

## 4. Discussion

The purpose of this preliminary study was to evaluate a sample of 30 amateur football players in order to establish possible relationships between the quality of movements and the different foot positions. Foot/ankle injuries were common in young male footballers. Some of the most common injuries are plantar fasciopathy, shin splints, muscular overloads, mainly of the posterior chain (triceps surae and hamstrings), and dynamic osteopathy of the pubis. These injuries are directly related to foot posture and incorrect biomechanics of the soccer player’s movement. Foot health professionals can intervene to improve stability and muscle control in soccer sports through muscle stretching, proprioception exercises, and foot core exercises [46]. Soccer is an explosive sport that executes sports gestures through important muscular forces. Strengthening the intrinsic musculature or the core of the foot by applying specific foot exercises complemented by the flexibility and movement capacity of other areas of the body, at the hip and abdominal level, can improve the quality of movement patterns and flexibility within myofascial chains. Although other studies such as Kim et al. and Sulowska-Daszyk et al. point out that a rigid deformity maintained for a certain period would not change [47,48], we believe that if a strong relationship between the two is established, a plan can be established to prevent foot injuries in amateur football players through podiatric control of foot posture. These health professionals can help develop prevention programmes to reduce injuries in soccer. The findings of this study are expected to provide valuable insights into the connection between movement quality and foot position among young non-professional football players. Understanding this relationship could help coaches, trainers, and healthcare professionals design more customised training programmes and injury prevention strategies. By identifying individuals with poor movement quality or atypical foot positions, targeted interventions can be implemented to address any underlying issues and reduce the risk of musculoskeletal injuries.

Numerous studies have investigated factors associated with sports injuries [46,49,50]. In 2015, Evans et al. evaluated 34 semi-professional soccer players for 12 months and reported a total of 273 soccer-related injuries, 114 involving the foot and ankle (70 ankles and 44 feet). The authors concluded that extensive studies should be conducted in non-professional soccer players to detect these risk factors for injuries and develop effective prevention advice [49]. However, the results showed that there is no statistical significance to demonstrate the relationship proposed in this novel study. According to the literature consulted, there are numerous studies that have applied both FPI and FMS in their studies in sports populations, obtaining beneficial results in their health [23,51,52,53,54,55,56]. However, there is a lack of studies investigating the relationship between FPI and FMS, making this research novel and significant.

Comparing this study with similar research is challenging since, to our knowledge, this is the first study to establish a relationship between foot posture and movement quality in amateur football players. However, we will analyse some of the similarities with previous studies, taking into account some of the differences such as terrain, land, and sport mode, among others [23,50]. Recently, the study by Castillo-Dominguez et al. analysed the relationship of the characteristics of the sole of soccer boots with the incidence of injuries in amateur players. The authors analysed a sample of 77 male soccer players who played 10 h a week. Their results were 41 injuries, which is equivalent to 1.81 injuries for each soccer player studied. These authors reported on the prediction of the sole characteristics with lesions in the lower extremities in soccer players. The variables of the soccer boot associated with the number of studs were associated with a higher prevalence of foot and ankle overload injuries [57].

Our FPI results showed similar overall mean scores of 0.96 (SD = 2.65) for the right foot and 0.90 (SD = 2.70) for the left foot. The foot posture of the right foot and the left foot presented a neutral position according to the FPI criteria defined by Redmon [33]. When comparing these results with the study by Cain et al. in a sample of 76 futsal players, where they obtained mean scores of 5.36 (SD = 2.92), it coincides that most of the athletes had a neutral or supinated foot position [52]. The pronated foot position was the least present in this type of athlete, and the pronated foot causes alterations in the gait cycle and can cause sports injuries [53]. In this line is the study by Cherati et al. who evaluated 68 futsal players with mean scores of 4.90 (SD = 2.88) for the right foot and 4.72 (SD = 3.08) for the left foot [23]. Both studies show differences contrary to our results.

In contrast, other authors, such as Lopezosa-Reca et al. compared football players and swimmers to determine the influence of sport type on foot positions by measuring FPI and knee Q-angle, obtaining normality scores with a mean FPI of football players of 2.23 (SD = 1.72) [54]. These results reflect similarities to the present study, as does the study by Sman et al. in Australian football and rugby players, where they found mean IPF scores of 3.3 (SD = 2.5). However, these comparisons have limitations due to differences in sport modes. Due to their intensity and torsion demands on the foot, these sports are particularly susceptible to ankle injuries, specifically ankle syndesmosis, with involvement of the distal tibiofibular joint ligament, the interosseous ligament, and the interosseous membrane, unlike soccer, where most studies indicate that the classic external ankle sprain injury is the most common in its presentation [58].

Considering that the relationship between foot morphology and injury is not clear, some studies point to the hypothesis that foot posture influences lower limb mobility and therefore injuries. The study by Torrotegui et al. carried out on a sample of 71 professional soccer players with a follow-up period of 3 years concluded that the playing position influences the incidence and severity of foot injuries, with the most numerous presentation in the thigh of the players. They also noted that increased exposure to training suggested a lower number of injuries [59]. This is the case of excessive foot pronation, which has been shown to be a risk factor for injury in some sports, such as football. Other studies indicate that the position of supinated feet means a higher risk of secondary injury from overuse, as occurs in sports gestures of football players [53]. This would imply its consideration as a risk factor for injury.

Other studies have combined the foot movement screen with other measures. For instance, the study of the Lisman et al., carried out on a cohort of 874 marine men. They observed that with a low running time and low FMS scores, the predictive value of getting injured increased. The authors correlated FMS with the physical aptitude test [60]. Koźlenia and Domaradzki’s study on a sample of 176 young athletes recommends routine training to improve movement patterns. The authors related the FMS to physical performance tests such as strength, power, flexibility, or balance. In our study, this test was related to the foot posture index, as it is a foot position test that considers three different foot positions that identify the morphology of the foot [61].

However, the results of the football players in this study for FMS showed a mean score of 14.30 (SD = 2.35), with a minimum score of 10 and a maximum score of 19. Most subjects had scores between 12 and 16. According to the recommendations of Cook et al., the results of this study carried out before the start of the competition season showed poor (53.3%) and optimal (46.7%) movement quality [18]. None of the athletes was able to achieve scores compatible with perfect movement quality. However, our results are consistent with those of Zalai et al., who obtained mean scores of 14.75 (SD = 1.51) in a sample of 20 professional football players in the preseason [62]. These authors found a direct relationship between different types of injury and the poor quality of certain FMS exercises. In particular, ankle injuries were associated with low values of the FMS hurdle step exercise, and knee and hip injuries with low values of the FMS deep squat exercise. Players who occupied the position of goalkeeper or defender showed better scores compared to the other positions in football. However, this investigation has not studied the position of goalkeeper.

In this sense, there is the possibility of a relationship between the playing position and movement quality and its implication and influence on the risk of injury in athletes. In 2022, a systematic review suggested that intervention programmes significantly improved total FMS scores in high-risk athletes [63]. In 2017, Dinc et al. evaluated 24 young football players to compare FMS scores before and after the exercise season, obtaining results similar to ours in the pre-season, of 14.83 (SD = 1.46), although these authors point out variations after exercise [36,64]. These authors noted variations in scores after exercise and suggested that exercise programmes aimed at limiting movement patterns are more effective than exercise programmes aimed at improving body control and coordination performance. However, the study by Rey et al. also used FMS in 23 football players to try to establish a relationship between the implementation of an injury prevention programme and the movement patterns of football players [65]. These authors concluded that such an injury prevention programme may not produce improvements in fundamental movement patterns in addition to a standard warm-up.

Despite the controversy surrounding the use of FMS as a method of detecting the risk of injury in football players, previous research suggests that higher scores would be associated with a positive impact on physical performance and, consequently, a lower risk of injury without physical contact, pointing to scores below 14 as situations that increase the risk of injury [15,66,67]. Recently, in 2022, Sikora and Linek suggested the need to use other tests that complement FMS to predict injuries in young football players [51]. However, this study was carried out with very young players and with few hours of training, compared to adult players as those in our study. We believe that to establish clearer relationships between foot position and movement quality in amateur footballers, larger studies with a larger number of variables, such as terrain or type of footwear, can influence subjects and possibly provide more scientific evidence for these relationships [68]. The type of footwear affects changes in the kinematics and kinetics of the joints of the lower extremities during sports practice and can affect performance in movements such as acceleration, braking, jumping, and/or turning. For example, the type of lacing, the number of eyelets, and the type of lacing can affect the stability of the foot and show different load indices and maximum pressure on the heel and/or metatarsals. Therefore, this will be a factor that will be developed in future studies.

We accept that there are limitations to this study. The results of this study should be understood as a pilot study with several limitations, starting with the relatively small sample size of the study. For future research, the population sample should be expanded to include female subjects, since women’s soccer is increasing in quantity and quality of female athletes and the incidence of knee injuries is higher. More research with larger sample sizes and a wider range of variables is needed to establish stronger relationships and generalise the findings. Additionally, future studies should consider the influence of the type of footwear on foot positions and movement quality. However, other similar studies with amateur football players employed a similar number of participants. A future study with a larger sample could establish stronger relationships and generalise its results.

## 5. Conclusions

There is no relationship between foot posture variables and analysis of movement quality measured with the FPI and FMS instruments. Therefore, we cannot demonstrate that foot posture is relevant in the quality of the movement of the football players studied. The results of this study may be useful in identifying possible foot posture problems that could affect the quality of the movement of players.

## Figures and Tables

**Table 1:** Frequencies and percentages of different foot positions and quality of the movements of soccer players.

Total Sample *n* = 30		Frequencies (*n*)	Percentages (%)
Foot Positions (FPI)	supination	11	36.7%
	neutral	15	50%
	Pronation	4	13.3%
Quality of Movements (FMS)	Poor quality	16	53.3%
	Good quality	14	46.7%
	Perfect quality	0	0%

**Table 2:** Cross-table of FPI and FMS of participants.

Total Sample *n* = 30	Quality of Movements (FMS)			
		Poor Quality (*n*–%)	Good Quality (*n*–%)	Perfect Quality (*n*–%)
Foot Positions (FPI)	Supination	6–20%	5–16.6%	0–0%
	Neutral	8–26.6%	7–23.3%	0–0%
	Pronation	2–6.6%	2–6.6%	0–0%

## Data Availability

The raw measurements are available by contact with the correspondence author.
